# Plants and Diabetes: Description, Role, Comprehension and Exploitation

**DOI:** 10.3390/ijms22083938

**Published:** 2021-04-11

**Authors:** Alessandra Durazzo, Massimo Lucarini, Antonello Santini

**Affiliations:** 1 CREA-Research Centre for Food and Nutrition, via Ardeatina 546, 00178 Rome, Italy; 2 Department of Pharmacy, University of Napoli Federico II, via D. Montesano 49, 80131 Napoli, Italy

**Keywords:** diabetes, medicinal plants, nutraceuticals, mechanism of action, markers

## Abstract

Many plants have been known for centuries to have medicinal importance with potential beneficial effects on health. Phytotherapeutic compounds are well known to play a globally significant role, in particular in the management and treatment of various chronic diseases. Among these, diabetes can cause long term damage to the body other than having a relevant economic burden on society being among the costliest chronic diseases. This motivated the focus of the proposed Special Issue, intended to develop and exploit the potential role of plants in the management and treatment of diabetes. The main topics included are: (i) description and use of medicinal plants for diabetes management; (ii) the elucidation and delineation of their main components, properties (anti-hyperglycaemic, hypoglicaemic, anti-infiammatory, apoptotic agents, etc.), (iii) the mechanism of action (in vitro and in vivo studies); (iv) formulation of nutraceuticals, botanicals, and dietary supplements useful as tools as an alternative or support to anti-diabetic pharmacological therapies; (v) development of new markers.

## Introduction

Many plants have been known for a long time to have medicinal values with potential beneficial effects on health [1,2,3,4,5,6,7,8]. There is a great research interest in better understanding diabetes and finding better treatment options, including the identification of natural products with anti-diabetic effects [9,10,11,12].

It is worth mentioning the recent work of Yeung et al. [13] which investigates the natural products in diabetes based on quantitative literature analysis and giving the main directions of natural product research in diabetes up to now and useful hints on promising avenues for future research. Some highly cited natural products or compound classes including curcumin, flavanone, resveratrol, carotenoid, polyphenols, flavonol, flavone and berberine have been considered.

Diabetes is a lifelong condition that causes a person’s blood sugar level to become too high. In recent years, diabetes prevalence increased in virtually all regions of the world, with millions of people worldwide now living with diabetes. This is a concern with deep effects on quality of life, demand on health services and economic costs. This health condition is related to a cascade of events which may include coronary heart disease, stroke and peripheral vascular disease, microvascular complications, renal disease, retinopathy and neuropathy, along with lower-extremity amputations [14].

With the growing rise in obesity and diabetes has come an increasing awareness of their impacts on infectious diseases, post-infection complications and mortality from critical infections implying also increased risk due to the coronavirus outbreak disease (COVID-19) that has become an evolving worldwide health crisis [15,16].

The focus of the proposed Special Issue is the potential role of plants in the management and treatment of diabetes. The main topics included are: (i) description and use of medicinal plants for diabetes management; (ii) the elucidation and delineation of their main components properties (anti-hyperglycaemic, hypoglicaemic, anti-infiammatory, apoptotic agents, etc.); (iii) their mechanism of action (in vitro and in vivo studies); (iv) the formulation of nutraceuticals, botanicals and dietary supplements; (v) the develop and novel anti-diabetic drugs; (vi) the development of new markers for this health condition.

At the interface between food and medicine, Jeremic et al. [17] showed the potential effects on metabolic syndrome of diallyl trisulfide derived from garlic and the effect on myocardial function in rats affected by metabolic syndrome. The topic was previously studied by the same authors [18] for its positive effects on the cardiovascular system in rats with chemically induced Type 1 diabetes mellitus.

Lu et al. [19] showed how a novel dipeptidyl peptidase IV inhibitory tea peptide improves pancreatic β-cell function and reduces α-cell proliferation in streptozotocin-induced diabetic mice.

In the scenario of the re-use of agro-food waste in the perspective of biorefinery and circular economy [20,21], the work of Giacometti et al. [22] showed a beneficial role of phenolic compounds from the olive leaf in the regulation of glucose homeostasis in the skeletal muscle, by improving glucose translocation and influence glucose uptake as the treatment for streptozotocin (SZT)-induced diabetes in rats.

It is worth mentioning the reviews on: (i) the role of isoflavones in type 2 diabetes prevention and treatment [23]; (ii) the therapeutic potential of apigenin [24]; (iii) multiple cell signaling pathways of human proinsulin C-peptide in vasculopathy protection [25]; (iv) the role of algae-derived components on Type 2 diabetes mellitus prevention and treatment [26].

This Special Issue’s end points have been to contribute to the growth of this area of research, trigger research interest on medicinal plants and diabetes and its implications, and reference their impact and use, by adding information scientifically substantiated with new data. We would like to thank all the Authors and the Reviewers of the papers published in this Special Issue for their great contributions and efforts. We are also grateful to the Editorial Board Members and to the Staff of the Journal for their kind support in the all the steps in the realization of this Special Issue.
